# Relationship between Height and Exposure in Multispectral Vegetation Index Response and Product Characteristics in a Traditional Olive Orchard

**DOI:** 10.3390/s24082557

**Published:** 2024-04-16

**Authors:** Carolina Perna, Andrea Pagliai, Riccardo Lisci, Rafael Pinhero Amantea, Marco Vieri, Daniele Sarri, Piernicola Masella

**Affiliations:** Department of Agricultural, Alimentary, Environmental and Forestry Sciences, Biosystem Engineering Division—DAGRI, University of Florence, 50144 Florence, Italy; carolina.perna@unifi.it (C.P.); andrea.pagliai@unifi.it (A.P.); riccardo.lisci@unifi.it (R.L.); rafael.pinheiroamantea@unifi.it (R.P.A.); marco.vieri@unifi.it (M.V.); piernicola.masella@unifi.it (P.M.)

**Keywords:** proximal sensor, precision olive growing, vegetation index

## Abstract

The present research had two aims. The first was to evaluate the effect of height and exposure on the vegetative response of olive canopies’ vertical axis studied through a multispectral sensor and on the qualitative and quantitative product characteristics. The second was to examine the relationship between multispectral data and productive characteristics. Six olive plants were sampled, and their canopy’s vertical axis was subdivided into four sectors based on two heights (Top and Low) and two exposures (West and East). A ground-vehicle-mounted multispectral proximal sensor (OptRx from AgLeader^®^) was used to investigate the different behaviours of the olive canopy vegetation index (VI) responses in each sector. A selective harvest was performed, in which each plant and sector were harvested separately. Product characterisation was conducted to investigate the response of the products (both olives and oils) in each sector. The results of Tukey’s test (*p* > 0.05) showed a significant effect of height for the VI responses, with the Low sector obtaining higher values than the Top sector. The olive product showed some height and exposure effect, particularly for the olives’ dimension and resistance to detachment, which was statistically higher in the upper part of the canopies. The regression studies highlighted some relationships between the VIs and product characteristics, particularly for resistance to detachments (R^2^ = 0.44–0.63), which can affect harvest management. In conclusion, the results showed the complexity of the olive canopies’ response to multispectral data collection, highlighting the need to study the vertical axis to assess the variability of the canopy itself. The relationship between multispectral data and product characteristics must be further investigated.

## 1. Introduction

Precision agriculture techniques are spread across all farming systems in various sectors, giving significant advantages in allowing higher production with higher sustainability [1,2,3,4]. Precision agriculture is based on data collection through multiple systems, such as remote sensing, unmanned aerial vehicles (UAVs), yield monitoring, weather stations, and ground-based sensors [5]. Those devices monitor and assess in-field variability, a critical element of precision agriculture management [6]. The olive growing sector is the most extended perennial growing in the Mediterranean basin, and it plays a fundamental economic and environmental role [7,8,9,10]. Still, this sector presents constraints and limitations due to structural characteristics and climate change [11]. Precision agriculture can be a viable solution to these problems [12,13]. In the olive growing sector, the application of sensor and precision agriculture techniques is already widely studied, with the vast majority of studies concentrating on using multispectral sensors mounted on UAV systems. UAV solutions are spread across all the sectors for precision agriculture data gathering, considering their availability, high-spatial-resolution data, and limited time consumption for in-field data gathering [14,15,16], giving optimum results for precision agriculture management in olive orchards [17]. However, UAV solutions (as well as satellite solutions) have some constraints in collecting data from canopies with vertical development, as they can only study the canopy from a top-down view [18], potentially losing information related to variability in the vertical axis of canopies. Consequently, it can be necessary to study the vertical axis separately using proximal sensors from the ground, particularly in traditional olive trees. Nonetheless, applying proximal sensors mounted on ground vehicles in the olive growing sector is still a subject scarcely investigated [19]. Still, it should be considered to better understand the behaviour of different parts of the canopies [20]. Canopy position can also affect olives’ and oils’ quality and quantity characteristics [21,22,23,24]. Considering those aspects, the present study has the following objectives:

1. To study on the vertical axis of olive tree canopies the effect of height and exposure on vegetation index results using a proximal multispectral sensor;
2. To investigate how height and exposure can affect the quantitative and qualitative characteristics of the olive product;
3. To study the relationship between vegetation indices obtained from a proximal multispectral sensor and olive product characteristics to assess whether this information can be useful for applying precision agriculture management strategies.

## 2. Materials and Methods

### 2.1. Experimental Site

The experimentation was carried out in the Ente Terre Regionali Toscane (Tuscany Region unit for agriculture) Centre for Innovation Testing and Transfer in Tenuta di Cesa farm, located in the municipality of Marciano della Chiana (Arezzo), in the centre-west of Tuscany, Italy (43°19′9″ N, 11° 47′51″ E, altitude 328.312 m AMSL).

The climate is classified in the Köppen and Geiger system as Csa, i.e., Mediterranean climate, characterised as being temperate and dry, with a hot summer. The average temperature of the coldest month is above 0 °C, with at least one month having an average temperature above 22 °C and at least four months having an average temperature above 10 °C. The mean annual temperature is 13° C, and the average yearly precipitation is 871 mm (Consorzio LaMMA c/o CNR-IBE Florence research area). The area is mainly identifiable as USDA Hydrologic Group C, with soils that present moderately high runoff potential when wholly wet, and water transmission through the soil is restricted.

**Figure 1:**
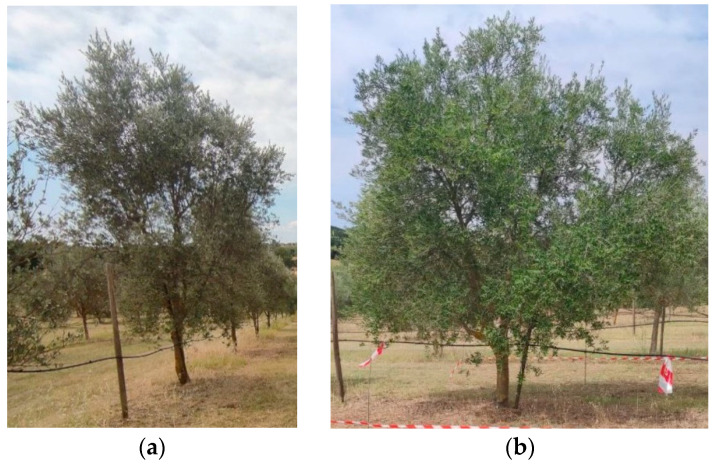
The two studied olives cultivars, Piangente (**a**) and Scarlinese (**b**).

An olive orchard was selected for the experimentation. The orchard was 1.95 ha, designed as a germplasm trail orchard, with five different cultivars (Pendolino, Colombino, Scarlinese, San Francesco, Piangente). The olive trees were planted in June 2009 with a 5 × 6 m planting layout; the primary orientation was north–south, and the plants were managed with a multi-branched (polyconic) vase pruning management (Figure 1). A drip irrigation system could allow the irrigation of the orchard, but it was not used during the experimentation season.

The results of a soil survey on electrical resistivity conducted in 2019 identified zones with similar resistivity, and site-specific soil samples and analysis were carried out for each zone. The experimentation site had a limited soil electrical resistivity variability with the following main characteristics: a medium clay content (41%), lower silt and sand content—33 and 26%, respectively; the pH was moderately alkaline (8.1), and the organic matter content was 2.53%. In general, no restrictions on macro- and micronutrients were present. The plants for the experimentation were selected within the same soil sub-zone.

A UAV flight performed by Pisa CNR allowed us to obtain a high-definition image of the experimental field and the digital terrain model (DTM) and digital surface model (DSM) of the fields. A DJI Phantom M4 (SZ DJI Technology Co., Ltd., Shenzhen, China) was used, mounted with a Six 1/2.9″ CMOS sensor: one RGB sensor and five monochrome sensors (spectral response in the blue, green, red, red edge, and NIR bands). Data were acquired from 11:30 to 12:30 on August 5th, with an 85% overlap in both directions. This allowed the creation of an orthophoto and a DSM, allowing us to measure the maximum height and area of the plants. The process followed for image elaboration is the same as in [25]. 

Six plants were selected for the characterisation: three plants of the Piangente (PT) variety and three of the Scarlinese (SC) variety. This way, three replicas for each cultivar were obtained. The two cultivars presented a different development, with the PT variety characterised by a vertically developed canopy. Instead, the SC plants were more horizontally developed (Figure 1a,b). The plants for each cultivar were selected for their similar canopy development and crop load. The six plants were divided into sectors, considering two heights and exposures. The heights, namely Top and Low, subdivided the plant into two sectors: from 0.90 m from the ground to 2 m (Low) and from 2 m from the ground to the maximum height (Top). The different exposures, namely East and West, subdivided the plants into two more sectors. So, a total of four sectors were created: Low–West (LW), Low–East (LE), Top–West (TW), and Top–East (TE). 

During the 2022 season, VI data were collected with a multispectral sensor mounted on a ground vehicle for each of the four sectors. In November 2022, a selective harvest was performed to harvest each plant and sector separately. At the time of the harvest, both quantitative and qualitative characterisation was performed. 

### 2.2. Vegetation Index Measurement

For the canopy and vegetation index (VI) characterisation at different heights and exposures, the OptRx^®^ crop sensor (AgLeader Technologies, Ames, IO, USA) was used. OptRx is an active sensor; i.e., it emits a light beam that hits the plants’ canopies, recording the response by measuring the reflectance in the 630–685 nm (red), 695–750 nm (RE—red edge), and 760–850 nm (NIR—near infrared) wavebands. The sensor stores canopy response wavelengths as points and automatically processes and calculates NDVI and NDRE indices using these wavebands. Each point measured by the sensor then contains information on the values of the three wavelengths and the vegetation indices. Those vegetative indices are obtained from the red, RE, and NIR wavebands with Formulas (1) and (2):

𝑁𝐷𝑉𝐼 = 𝑁𝐼𝑅 − 𝑅𝐸𝐷/𝑁𝐼𝑅 + 𝑅𝐸𝐷(1)

𝑁𝐷𝑅𝐸 = 𝑁𝐼𝑅 − 𝑅𝐸/𝑁𝐼𝑅 + 𝑅𝐸 (2)

OptRx was developed for open-field crops (such as wheat or corn); consequently, this sensor is usually placed parallel to the ground at an average height of 1.2 m from plant crowns. For this experiment, however, the sensor was placed perpendicular to the ground and parallel to the crown of the plants. Therefore, it was deemed necessary to evaluate the correct positioning of the sensor. In particular, it was necessary to determine at what distance from the canopies the sensor had to be placed to acquire data, as this distance is a crucial element for proper data collection, as suggested by [26]. Different positions were evaluated from a literature review, as shown in Table 1.

**Figure 2:**
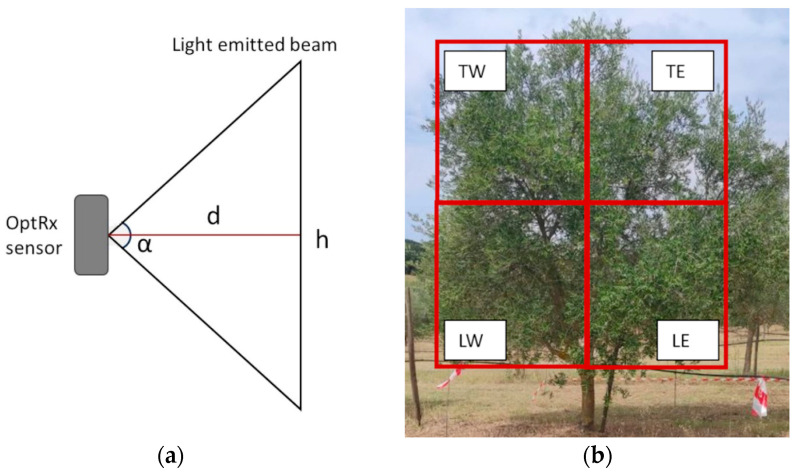
Schematic representation of the light beam measure (**a**) and schematic representation of the canopy subsetting based on the exposure and the height, namely TW: Top–West, TE: Top–East, LW: Low–West, and LE: Low–East (**b**).

Considering the various positions employed by the previous studies, it was deemed necessary to evaluate, for proper sensor placement, the height of the light beam emitted at different distances from a target. For this evaluation, the sensor was placed on a pole in a dark room in front of a smooth white wall at four distances (d)—1.5, 1.2, 0.9, and 0.7 m. For each distance, the beam height (h) was measured. (Figure 2a). For each measurement, the orthogonal position of the sensor relative to the target was evaluated, and the symmetry of the emitted light beam was assumed. The angle (α) between the emitted light beam’s starting point and the light beam’s height for each distance from the target was obtained by a simple trigonometric calculation. The measure of the sensor’s emitted light beam resulted in a mean angle between the light beam height and the distance of the sensor from the objective of 120°. Based on the result, it was possible to choose the sensor setting, as explained below. 

**Figure 3:**
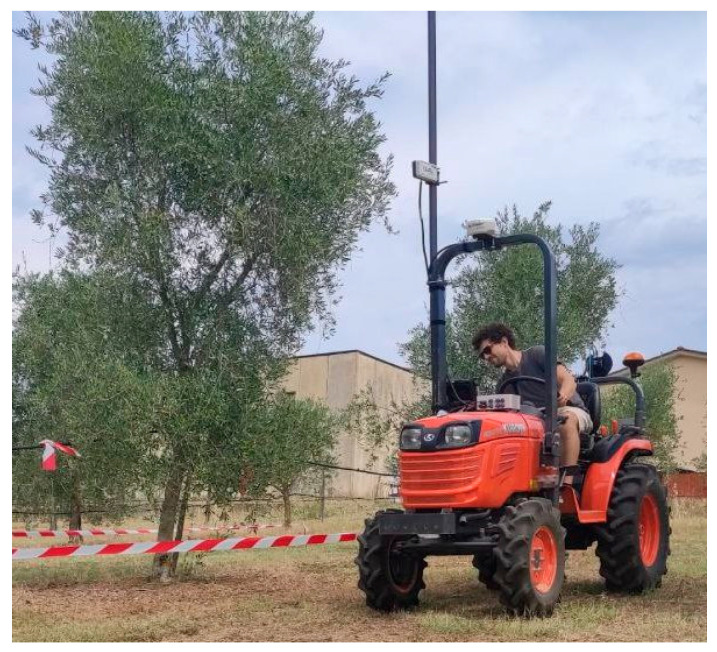
Multispectral data collection setting: on the roll-over structure is positioned the GNSS receiver; on the left is it possible to see the steel pole on which the multispectral sensor is mounted. The red and white strip allowed the same distance from the plants to be maintained for each replication.

The multispectral sensor was mounted on a ground vehicle, a Kubota B2420 tractor (Kubota, Osaka, Japan), to collect data automatically. The sensor was paired with a GNSS receiver, GPS 6500 from AgLeader Technology (Ames, IO, USA), mounted atop the tractor’s roll-over protective structure (ROPS), allowing a georeferentiation for every point collected by the OptRX sensor. The instrumentation was coupled with the hardware and the rough book (Panasonic ToughPad FG-Z1, Panasonic Corporation, Kadoma, Japan) for data collection and storage, and the point data were stored in .csv format. A 3 m graduated steel pole was installed on the side of the ROPS structure to collect data at different heights (Figure 3). The sensor was placed 1.6 and 2.5 m from the ground, and the tractor maintained a 1 m distance from the canopies. The distance was chosen from the results of the light-emitted beam height and angle from the objective as described before. This way, the emitted light beam height was 1.2 m, which was considered optimal for assessing the entirety of the canopy through the two different measures. This way, the assessment of the VIs at two heights corresponded to the same partition of the plants as described before (Figure 2b).

Three replications were collected for each height, exposure, and plant. To maintain the same distance from the canopies for each replication, a signal (i.e., a red and white strip tied to two poles) was placed 1 m from the most overhanging part of the canopy, indicating the correct path. The multispectral sensor, coupled with the GNSS receiver, collected and recorded data every 0.2 m, and the tractor maintained an average speed of 1.38 m. As a result, the points collected for each plant differed according to canopy length. Data were collected on August 8, at noon, to limit direct canopy irradiance.

### 2.3. Qualitative and Quantitative Product Characterisation

Selective harvesting was carried out in November 2022. Each selected plant and sector was harvested individually with a manual shaker. Before harvesting, 100 olives per sector and plant were randomly selected; on that sample, the following measures were taken: resistance to detachment (RtD), measured with a dynamometer (0–1000 g, ARPO, France); diameter of the fruit’s central section (Dmt), measured with a digital caliper (range 150 mm, resolution 0.01 mm) (METRICA, Milan, Italy); hardness of the fruit’s epicarp (Dur), measured with a durometer (type O, Shore hardness measuring scale 0–100); maturation index, measured following [35]; and weight of 100 olives (OW) measured with a digital scale (KERN&SOHN digital balance, Balingen, Germany). A total of 300 samples per sector per cultivar were analysed this way.

After the sampling, all the sectors and replicates were completely harvested, and the weight of total olives per sector (ToW) was measured and collected for oil extraction. 

It was possible to obtain a sample of olive oil from each sector for each plant through micro-milling and microextraction. Studies by [36,37] were followed for the microextraction procedure. The procedure consisted of a crusher, a lab-scale malaxator, and a laboratory centrifuge (NEYA8, Neya centrifuges, Modena, Italy). After crushing, the total olive paste obtained from each sample was mixed in a hermetically sealed cylindrical malaxator for 30 min, maintaining the temperature of the paste at 23 °C. Then, the paste was centrifugated for 10 min at 6500 r.p.m. and recovered with a separatory glass funnel. In this way, centrifugation separated the oil from vegetation water and solid fractions. After extraction, the yield (Y) of each sample was measured and expressed as the percentage of oil weight over olive paste weight. The samples were analysed by ISVEA s.r.l. laboratory (Istituto per lo Sviluppo Viticolo Enologico ed Agroalimentare, Poggibonsi, Italy). Free fatty acid content (FA) (by regulation: Reg CEE 2568/1991 11/07/1991 GU CEE L248 05/09/1991 All II Reg UE 2016/1227 27/07/2016 GU UE L202/7 28/07/2016 All I), number of peroxides (Prx) (by regulation: Reg CEE 2568/1991 11/07/1991 GU CEE L248 05/09/1991 All III Reg UE 2016/1784 30/09/2016 GU UE L273/5 08/10/2016 All), and total polyphenols (Plyph) (laboratory’s internal system of analysis: ML025 rev. 0 2006) were analysed. A total of 3 samples per sector per cultivar were analysed this way.

### 2.4. Statistical Analysis

VI and product characterisation data were stored in Excel sheets (Microsoft CO, Washington, DC, USA). For VIs, cleaning the data from outliers or wrong values was necessary. The multispectral data were in point form, with each point characterised by its spatial location and the measured wavelength and VIs. Points with negative wavelength values corresponding to empty areas (i.e., measurements taken between plants) were considered erroneous, and values exceeding the VI threshold referring to vegetation (from 0 to 1) were considered outliers. After the cleaning, the multispectral sensor data were sampled through GIS software (QGis 3.18 Zürich). One hundred values of the two VIs were extracted for each sampled plant for every sector, creating a sample of 300 points per sector per cultivar. After the sampling, statistical analysis was performed using RStudio (version 2023.06.0 Build 421) [38]. Some transformations were needed for olives’ and oils’ qualitative and quantitative characterisation variables; logarithmic and Tukey transformations were performed to achieve data normalisation. Tukey’s test for multiple comparisons was used to estimate the difference between all parameters considering the effect of the different heights, exposures, and cultivars, with a total of eight estimates. Bonferroni confident level correction was applied to Tukey’s test, and *p*-values were considered significant at α = 0.05. To apply the test, the “emmean” R package was used. Linear regressions were performed to evaluate the relationship between the two VIs and the product characteristics. To create the linear regression, the mean values of all the parameters collected per sector and plant (i.e., a total of 12 values per parameter per cultivar) were used. The results were plotted in a linear regression plot, and the R^2^ value and intercept were computed. The confidence band, highlighting the confidence interval of 95% of the regression line, was also estimated. To create the graph and calculate the R^2^ and the intercept, the “ggplot2” RStudio package was used. 

**Figure 4:**
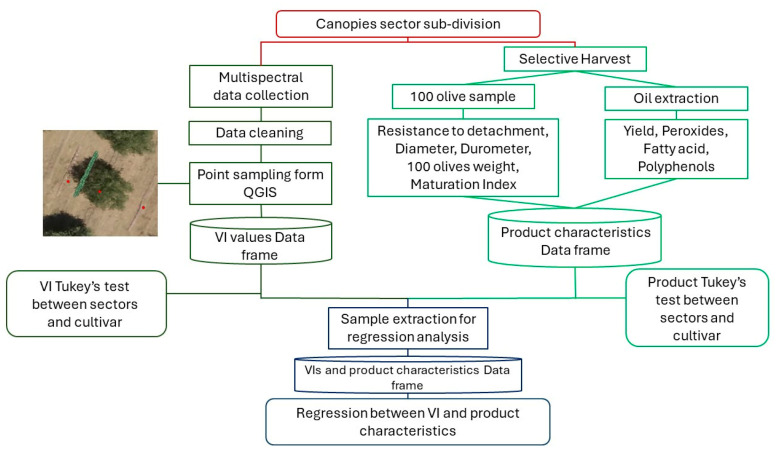
Flow chart of the applied methodology for data collection and data analysis. In red, the starting point of data collection; in dark green, the workflow for VI data; in light green, the workflow for product data; in dark blue, the process for regression study between VIs and product characteristics.

The process is summarised in the flow chart in Figure 4.

## 3. Results

### 3.1. Plant Dimensions

Through the UAV data, specifically the orthophoto reconstruction and the DSM computing, it was possible to obtain the maximum height and area of the six selected plants. Data are shown in Table 2.

It was possible to see that the PT variety tends to have taller plants with limited horizontal canopy development; instead, the SC plants had more evident horizontal development and height similar to the PT. Consequently, the positioning at the two different heights could be considered representative for both cultivars.

### 3.2. Vegetation Index Results

**Figure 5:**
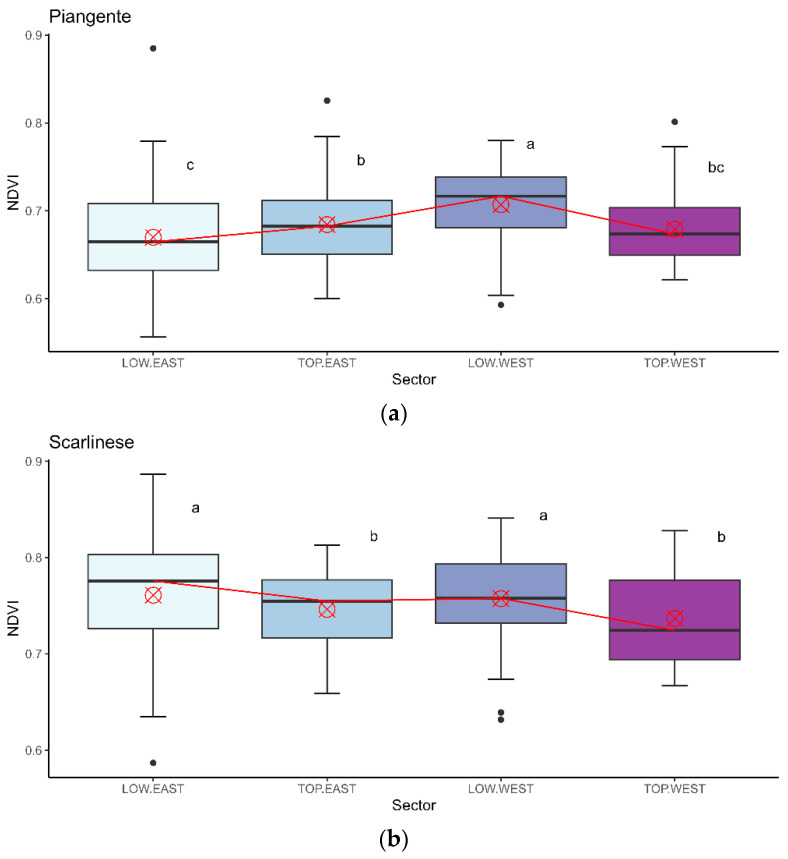
Boxplot for the different sectors and cultivars for the NDVI index. In (**a**), the PT cultivar’s data are presented; in (**b**), the data for the SC cultivar are presented. The red cross specifies the mean positioning, and the red line defines the difference in positioning between the various medians, highlighting the variation in median values between the sectors. The letters near the boxplots state the differences between the values; different letters indicate a statistically significant difference (*p* ≤ 0.05) in Tukey’s test.

**Figure 6:**
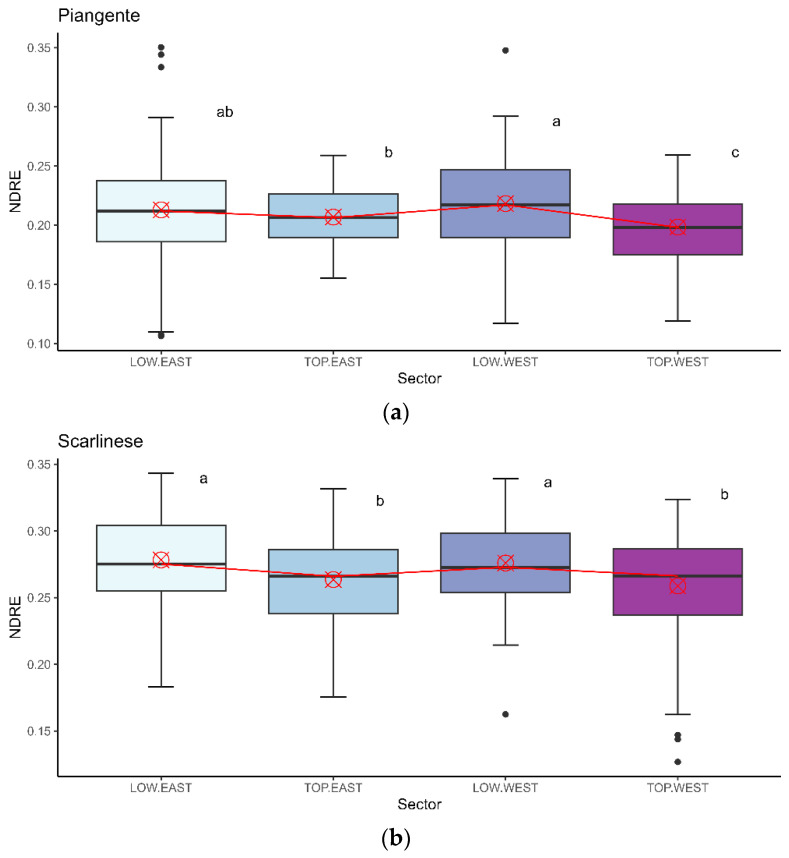
Boxplot for the different sectors and cultivars for the NDRE index. In (**a**), the PT cultivar’s data are presented; in (**b**), the data for the SC cultivar are presented. The red cross specifies the mean positioning, and the red line defines the difference in positioning between the various medians, highlighting the variation in median values between the sectors. The letters near the boxplots state the differences between the values; different letters indicate a statistically significant difference (*p* ≤ 0.05) in Tukey’s test.

Multiple comparisons between the different sectors and cultivars for the VI data highlighted different patterns, particularly between the cultivars. In Figure 5 and Figure 6, the boxplot graphs for the two VIs show the results for each sector, where the two cultivars are evaluated separately.

Considering the NDVI for the PT plants (Figure 5a), it was possible to see median values varying around 0.675 and 0.775, with the highest median value registered for the LW sector. Some differences could be determined: NDVI values significantly differed between the LW and LE areas, with LE recording higher values than LW. In the West exposure, values in the Low areas were statistically higher than those in the Top sectors, but in the East exposure, an inverse trend can be seen, with Low values lower than Top values. Consequently, the influence of exposure and height could be observed. For plants of the SC cultivar (Figure 5b), the median values of the NDVI index were slightly higher than those of the PT, extending from 0.725 to 0.785. The LE sector displayed the greatest variability. Multiple-comparison analysis showed significant differences between the heights, with higher values in the Low sector than in the corresponding Top sector. In this case, no significant differences were observed between the exposures, although the values recorded for the East exposure at both heights were slightly higher than those for the corresponding West exposure.

Different trends were observed in the NDRE index. Compared with the NDVI results, for NDRE, the values aggregated around a narrower range in both cultivars: for the cultivar PT (Figure 6a), median values ranged from 0.200 to 0.225, and values fluctuated around the threshold of 0.255 for the cultivar SC (Figure 6b). There were no statistical differences between LW and LE for the cultivar PT, which had values between 0.12 and 0.35 in both cases. The Top sector had lower values than the corresponding Low sector, and TE recorded higher values than TW. Consequently, a height effect was present for the NDRE of the cultivar PT (Low values higher than Top values). For the cultivar SC, the distribution pattern of NDRE was identical to that of NDVI for the same cultivar: the Low sectors showed higher values than the corresponding Top sectors, and the East exposure recorded slightly higher values than the West exposure. However, the statistics did not confirm the latter difference.

### 3.3. Qualitative and Quantitative Product Characterisation Results

Table 3 and Table 4 show the mean values and standard deviation of the quantitative and qualitative characteristics of olives and oils and Tukey’s test results, divided among different sectors and cultivars. The Dmt measurement presented a substantial difference between the cultivars; PT recorded bigger fruits, with diameters ranging between 14 ± 1 mm. SC registered dimensions ranging between 10 ± 1 mm. Additionally, in both cultivars, the Top sectors registered significantly higher Dmt values than the corresponding Low sectors. Exposure, on the contrary, seemed to have no effect. The RtD variable showed differences among both cultivars and sectors. Cultivar PT had lower RtD values, ranging from 380 to 460 g; SC, on the other hand, recorded values from 510 to 560 g. RtD was statistically higher for each cultivar in the Top sector. Exposure seemed to have a weaker effect on the variable, although it was possible to observe that PT values were slightly higher in the East exposure than in the West exposure. However, these differences were not validated by statistics. 

No particular differences were obtained for the Dur variable. PT registered values ranging from 38 to 42 on the Shore scale. Instead, SC registered values from 40 to 42. Tukey’s test did not highlight any significant differences. Values of the ToW variable were substantially higher for the cultivar SC (4500–7000 g) and lower for PT (900–3000 g). There were no statistically significant differences between the sectors, although the Top sectors recorded higher values than the corresponding Low sectors in both cultivars. For SC, East exposure recorded higher values than West exposure. OWs showed substantial differences between cultivars, with PT recording higher values than SC (260–280 and 122–137 g). Although there were no significant differences between the sectors, for both cultivars, the Low sector values were slightly higher than the corresponding Top values. This trend was similar to that of the Dmt variable. MI results were statistically different among cultivars but were homogeneous across sectors, and no clear patterns or statistical differences were present. The differences between cultivars showed a higher degree of maturity for SC than for PT, with the former obtaining values around 4 and the latter closer to 2.

FA content values of the extracted olive oil still did not show significant differences between the sectors but did show significant differences between the cultivars. The PT cultivar recorded values between 0.23% and 0.30% oleic acid, and the SC variety between 0.14% and 0.15%. For Prx content, the results were not statistically different, with the PT cultivar raging between 5.5 and 6.8 Meq O_2_ Kg^−1^ and SC between 4.8 and 6.0. It was possible only to identify slightly higher values for the East exposure, but they were not statistically significant. Regarding Plyph values, statistically significant differences were obtained only between cultivars. PT recorded higher values, ranging from 1012 to 1068 mg Kg^−1^ gallic acid, compared to SC, which recorded 443 to 561 mg Kg^−1^ gallic acid. Y results differed substantially between cultivars, with PT recording higher values (10.9–13%) than SC (4.23–4.98%). The cultivar PT obtained results in Y slightly higher for East exposure than for West, but statistics did not confirm the difference. This trend was not present for the SC cultivar, which recorded more similar values among the sectors.

### 3.4. VI and Product Characterisation Regression

**Figure 7:**
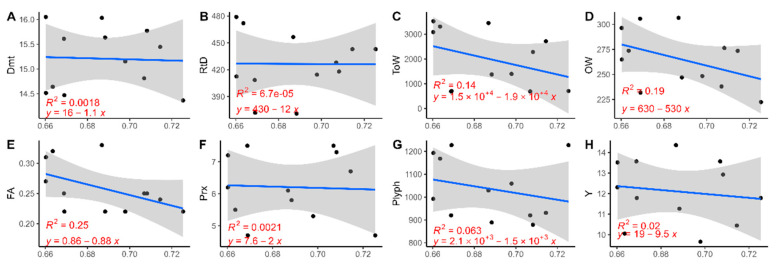

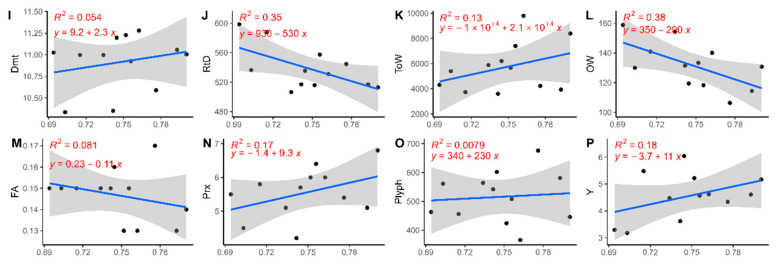
Scatter plot and linear regression for the NDVI and the product characterisation variables for the PT cultivar (**A**–**H**) and the SC cultivar (**I**–**P**). The blue line represents the regression line; the grey area represents the confidence band (95% confidence interval).

Figure 7 shows the linear regressions for cultivars PT and SC for the relationship between NDVI and product characterisation. Figure 7A–H expose the results for PT. It was possible to assess the absence of a relationship for each variable studied, as the R^2^ values recorded were too low to state a significative relationship. Only in the relationship between NDVI and FA (Figure 7E) was it possible to see an R^2^ = 0.25, still a nonsignificant value. A negative trend was present in all relationships in the cultivar PT, particularly for the variables ToW, OW, and FA. In the case of SC (Figure 7I–P), the R^2^ values were not statistically significant. RtD and OW (Figure 7J,L) had R^2^ values greater than 0.30 (R^2^ = 0.35, R^2^ = 0.38), and the trend was negative in both cases. 

**Figure 8:**
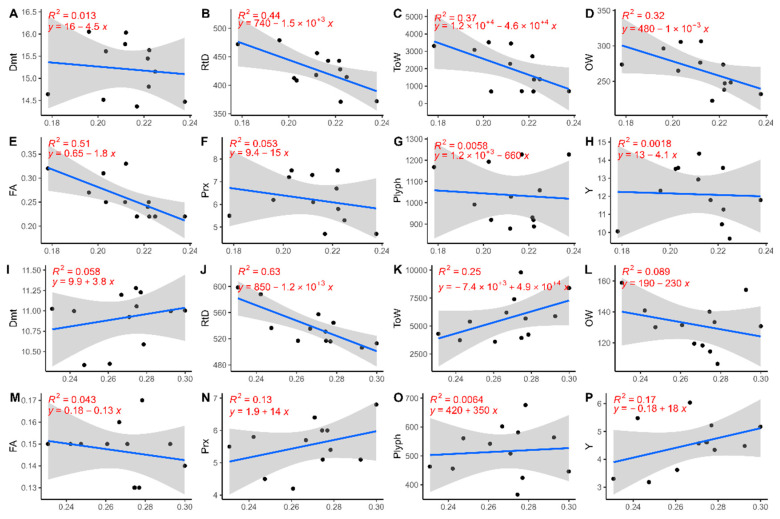
Scatter plot and linear regression for the NDRE and the product characterisation variables for the PT cultivar (**A**–**H**) and the SC cultivar (**I**–**P**). The blue line represents the regression line; the grey area represents the confidence band (95% confidence interval).

Figure 8 shows the linear regression for cultivars PT and SC for the relationship between NDRE and product characterisation. Again, highly significant R^2^ values were not obtained, although the regression values were slightly higher for some variables than those measured for NDVI relationships. For PT, R^2^ values greater than 0.35 were recorded for RtD, ToW, and FA variables (R^2^ = 0.44, R^2^ = 0.37, and R^2^ = 0.51) (Figure 8B,C,E). For all these variables, the trend was negative, confirming the trend observed for the NDVI study. For NDVI and NDRE in cultivar PT, the linear regression for Plyph, Prx, and Y was essentially insignificant. RtD linear regression (Figure 8J) measured a good R^2^ value (R^2^ = 0.63) with a negative trend for the cultivar SC. The R^2^ values were too small for the other variables to assess trends. 

**Figure 9:**
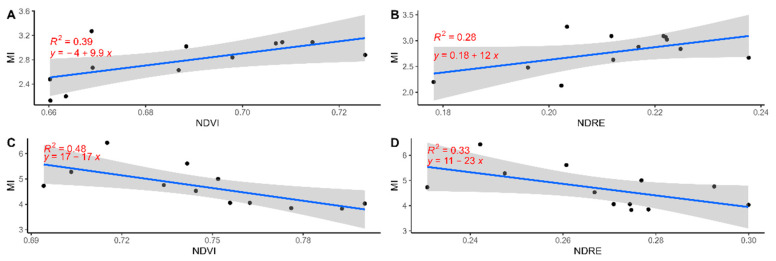
Linear regression and relationship for the two VIs and the MI values for the PT cultivar (**A**,**B**) and the SC cultivar (**C**,**D**). The blue line represents the regression line, and the grey area represents the confidence band (95% confidence interval).

Figure 9 shows the relationship between the two VIs and the MI variables for PT (Figure 9A,B) and SC (Figure 9C,D). No significant R^2^ values were obtained, with the highest value being for NDRE vs. MI for the SC cultivar (R^2^ = 0.48) (Figure 9C). Nonetheless, two distinct trends were observed: for PT, the regression was positive; instead, the trend was negative for SC.

## 4. Discussion

Vegetation indices based on canopy reflectance are, to date, one of the key elements for the application of variable-rate management practices and precision agronomic management of fields and orchards. However, the application of these indices and technologies has not yet been implemented in the traditional olive sector. This may be due, in part, to the complexity of olive tree canopy development and characteristics, elements that can influence canopy reflectance and, consequently, vegetation index responses [39]. Taking into account this element and the variability of the product characteristics and its distribution in different canopy sectors [40], the present study had two main objectives: (a) to study through a multispectral sensor the response of vegetation indices of the canopies of two olive cultivars at different heights and exposures and, at the same time, to characterise the product in the same different heights and exposures and (b) to study the possible relationships between VIs and product characteristics. For this purpose, a ground vehicle with a georeferenced proximal multispectral sensor was deployed, and data from two VIs (NDVI and NDRE) were collected; canopy responses to the sensor were studied in four sectors according to height (0.9–2 m, 2 m–max canopy height) and exposure (West or East). The behaviour of VIs in the different sectors showed for each index, sector, and cultivar—except for the East exposure in PT—higher values for both VIs in the lower part of the canopies than in the corresponding upper part. For the present study, the authors investigated if there were differences in the VI responses at different heights for PA management purposes: the results showed that applying just one height when using a multispectral sensor on the vertical axis of the canopy can be reductive, as it cannot show all the variability thought all the canopy. This can lead to different needs for the canopies in terms of localised fertilisation and canopy biomass management through pruning.

The product was selectively harvested based on the same four sectors applied in the VI data collection. The obtained results confirmed what previous studies found. The higher values obtained for both the total weight (ToW) and the 100-olive weight (OW) for the upper part of the canopy follow the trend seen in previous research [40,41,42]. This behaviour may be due to the influence of higher irradiance in the upper part of the canopy, resulting in increased fruit size and weight [23,43]. This also justifies the behaviour of olive size (Dmt), which is larger in the upper part of the canopy. In the case of resistance to detachment (RtD), the influence of the cultivar is the main aspect to consider [44]. However, other elements should also be considered: different irradiation levels may have an effect [40], leading to a higher level of RtD with higher irradiation. Maturation index (MI) and epicarp resistance to penetration (Dur) do not differ significantly between sectors, and only the effect of the cultivar should be taken into account. 

In oils’ qualitative characterisation analysis, particularly for the peroxide content, a minor but nonsignificant difference was obtained between the sectors. This trend was also obtained in previous research studying cultivars Arbequina, Manzanilla de Sevilla, and Manzanilla Cacereña [40,41,42]. In the case of fatty acids, the lack of difference between sectors confirms previously studied trends [45]. In the case of yield, the results were similar between the sectors, with only slightly higher values in the upper part of the canopy, similar to a previous study [41].

The results of the present study show that although product quality is not strictly dependent on the positioning of fruit plants, certain elements are, particularly RtD, Dmt, and ToW. These elements are of particular interest because they influence harvest efficiency [46,47].

A linear regression was performed between the VI and product data to assess the possibility of a relationship between these variables. The results showed no or poor relationships between the parameters. Insignificant R^2^ values were obtained, with a maximum value of R^2^ = 0.63, still a nonsignificant value. However, it was possible to evaluate some trends, particularly between RtD and NDRE values for both cultivars (R^2^ = 0.63 for PT and R^2^ = 0.44 for SC). The results showed a negative trend, i.e., a lower RtD value in the presence of higher VI values, suggesting that a more vigorous area may reduce the fruit detachment strength. Another noteworthy feature is the different responses of the two cultivars for some regressions. In particular, an inverse trend can be seen in the MI and ToW regressions: in the first case, PT had a positive trend, while SC obtained a negative trend. In the second case, ToW, cultivar PT obtained a negative trend. On the other hand, SC showed a positive trend, confirming the results of previous studies [48,49]. The results show that the effect of cultivar must be considered in the use and study of VIs in olive grove management. Cultivars can strongly influence the response and behaviour of VIs [50], the product, and, consequently, their interaction.

## 5. Conclusions

Few studies have used a ground-mounted proximal multispectral sensor in olive growing. To address differences in the response of vegetation indices, it is more common to use a UAV-supported sensor. However, this type of data collection may lose information on the vertical axis of canopies. The present study aimed to investigate the effect of height and exposure on the response of vegetation indices, detected by a proximal multispectral sensor, and product characteristics for two olive cultivars. The results show that height can affect the VI response, which means that studying the vertical axis of the canopy by applying only a height to the sensor may be reductive. In particular, the results showed higher VI values in the lower part of the canopies. However, further studies are needed. In particular, only one type of exposure (west–east exposure) was analysed in the present study, but a north–south exposure study should be considered to confirm this trend. In addition, the study evaluated only two cultivars, and considering the high variability among cultivars in the results obtained, it is necessary to assess the behaviour of other olive varieties. However, knowing that plants can have different results regarding vegetation index and vigour status at different heights can be valuable information for managing olive groves with precision agriculture techniques.

The product characterisation showed little height and exposure effects, with some exceptions. In particular, for some characteristics (fruit resistance to detachment, diameter, and total harvest weight) that can affect harvest efficiency, the results were statistically higher in the upper part of the canopies. The linear regression between the vegetation indices and the product characteristics did not show significant values, just some trends, as in the case of the negative relationship between the NDRE and the fruit resistance to detachment (R^2^ = 0.44–0.63). More studies are needed to assert with a reasonable degree of certainty the relationship between VIs and olive production, particularly between the VIs and the product characteristics that can influence harvest efficiency. Nonetheless, other elements should be considered in investigating these relationships; in particular, the cultivar effect should be replicated, and the seasonal and temporal impact should also be examined.

## Figures and Tables

**Table 1:** Literature references for sensor positioning classified based on the distance from the objective, the different heights, and the kind of cultivation on which the measurements were performed.

Reference	Distance from Canopy	Crop
[27]	80 cm	Vine
[28]	50–80 cm	Potato
[29]	1.2–1.6 m	Winter wheat
[30]	≈1 m	Peach
[31]	0.75 m above the ground (about 0.5 m above the pasture, considering an average pasture height of 0.25 m)	Pasture
[32]	0.6–0.7 m	Peanut
**Reference**	**Different Heights**	**Crop**
[33]	3, but not specified which	Lab test, no crop specified
[34]	0.8, 1.6, and 2.4 m from the ground	Apple

**Table 2:** Height and area calculated from UAV data.

Cultivar	Plant Number	H Max m	Area Max m^2^
PT	1	4.00	5.39
	2	4.11	4.99
	3	3.75	6.30
SC	4	3.60	12.50
	5	3.75	15.40
	6	3.13	9.69

**Table 3:** Mean and standard deviation for olives’ characterisation variables: fruit diameter expressed in mm, resistance to detachment expressed as gf, fruit resistance to penetration expressed as Shore hardness scale value, total harvest weight expressed in g, hundred olives’ weight expressed in g and maturation index expressed in a scale from 0 to 7, where 0 indicates not ripe and 7 extremely ripe. The values are classified based on the cultivar (PT and SC) and the sectors (LE, LW, TE, and TW). The letters between the brackets state the differences between the values; different letters indicate a statistically significant difference (*p* ≤ 0.05) in Tukey’s test. ^1^ Is the mean value of 300 samples. ^2^ Is the mean value of 3 samples.

		Dmt ^1^	RtD ^1^	Dur ^1^	ToW ^2^	OW ^2^	MI ^1^
		µ ± σ	µ ± σ	µ ± σ	µ ± σ	µ ± σ	µ ± σ
PT	LE	14.78 ± 1.08 (b)	428.50 ± 144.33 (ef)	38.03 ± 8.00 (c)	923.06 ± 391.38 (c)	261.54 ± 38.96 (a)	2.986 ± 0.301 (a)
	LW	15.24 ± 1.12 (b)	383.83 ± 166.10 (f)	40.28 ± 7.68 (abc)	930.07 ±403.51 (c)	236.30 ± 13.05 (a)	2.93 ± 0.122 (a)
	TE	15.38 ± 1.37 (a)	464.67 ± 154.81 (d)	42.88 ± 8.12 (ab)	3084.99 ± 695.70 (bc)	282.60 ± 21.47 (a)	2.616 ± 0.48 (a)
	TW	15.40 ± 1.41 (a)	429.01 ± 160.16 (de)	41.03 ± 8.10 (ab)	3035.88 ± 299.53 (bc)	281.21 ± 13.13 (a)	2.59 ± 0.455 (a)
SC	LE	10.80 ± 0.82 (d)	513.50 ± 126.48 (bc)	41.49 ± 9.77 (bc)	6006.81 ± 2146.18 (ab)	122.40 ± 13.87 (b)	4.386 ± 0.778 (b)
	LW	10.64 ± 0.70 (d)	531.34 ± 126.44 (c)	42.31 ± 9.09 (ab)	4471.79 ± 1230.90 (ab)	133.36 ± 19.99 (b)	4.733 ± 0.89 (b)
	TE	11.17 ± 0.84 (c)	549.33 ± 154.10 (a)	40.56 ± 10.41 (a)	6976.11 ± 3055.33 (a)	133.09 ± 12.83 (b)	4.85 ± 1.368 (b)
	TW	11.07 ± 0.84 (c)	558.83 ± 136.48 (ab)	42.14 ± 10.62 (a)	5393.84 ± 979.03 (a)	137.23 ± 19.96 (b)	4.753 ± 0.235 (b)

**Table 4:** Mean and standard deviation for oils’ characterisation variables: fatty acids expressed as percentage of oleic acid, peroxide content expressed as milliequivalents of O_2_ Kg^−1^, polyphenols expressed as mg Kg^−1^ of gallic acid, and yield expressed as percentage of extracted oil. The values are classified based on the cultivar (PT and SC) and the sectors (LE, LW, TE, and TW). The letters between the brackets state the differences between the values; different letters indicate a statistically significant difference (*p* ≤ 0.05) in Tukey’s test. Each value for the parameters is the mean value of 3 samples.

		FA	Prx	Plyph	Y
		µ ± σ	µ ± σ	µ ± σ	µ ± σ
PT	LE	0.230 ± 0.017 (a)	6.000 ± 1.411 (a)	1012.00 ± 186.840 (a)	12.210 ± 1.206 (a)
	LW	0.230 ± 0.017 (a)	5.833 ± 1.474 (a)	1068.667 ± 153.728 (a)	11.673 ± 1.958 (a)
	TE	0.297 ± 0.042 (a)	6.867 ± 0.666 (a)	1033.667 ± 157.052 (a)	13.603 ± 0.719 (a)
	TW	0.277 ± 0.040 (a)	6.133 ± 0.603 (a)	1030.33 ± 123.062 (a)	10.940 ± 1.202 (a)
SC	LE	0.153 ± 0.015 (b)	5.567 ± 1.159 (a)	561.00 ± 115.00 (b)	4.230 ±1.000 (b)
	LW	0.143 ± 0.012 (b)	4.80 ± 0.52 (a)	562.333 ± 19.553 (b)	4.237 ± 0.538 (b)
	TE	0.143 ± 0.012 (b)	6.067 ± 0.306 (a)	443.333 ± 71.842 (b)	4.890 ± 0.512 (b)
	TW	0.147 ± 0.015 (b)	5.733 ± 0.252 (a)	496.333 ± 93.565 (b)	4.853 ±1.406 (b)

## Data Availability

Dataset available on request from the authors.
